# Monkeypox outbreak: A novel threat after COVID-19?

**DOI:** 10.1186/s40779-022-00395-y

**Published:** 2022-06-13

**Authors:** Yang Zhang, Ji-Yuan Zhang, Fu-Sheng Wang

**Affiliations:** Senior Department of Infectious Diseases, the Fifth Medical Center of Chinese PLA General Hospital, National Clinical Research Center for Infectious Diseases, Beijing, 100039 China

**Keywords:** Monkeypox, Outbreak, Zoonosis, Human-to-human transmission

Dear Editor,

Monkeypox is a zoonosis caused by monkeypox virus (MPXV) infection first reported in Central and West Africa [1]. The first outbreak of monkeypox outside of Africa was reported in 2003 [2]. After that, Israel, the United Kingdom (UK), Singapore, and other countries have reported monkeypox cases among travelers back from Nigeria since 2018 [3]. On May 7, 2022, the UK Health Security Agency (UKHSA) reported the first monkeypox case in this outbreak. The patient developed a rash in Nigeria on April 29 and was confirmed as monkeypox in London [4]. On May 14, two additional cases living in the same household were diagnosed with monkeypox in London. On May 15, the UKHSA reported another four cases with no history of contact with the three cases previously identified [4]. Common contacts have been identified for two of the four new cases, suggesting a community transmission of the MPXV in the UK [5]. Subsequently, increasing cases of monkeypox have been reported in other non-endemic countries, mainly in Europe and North America [6] (Additional file 1: Fig. S1). As per the latest reports from the Monkeypox Tracker Dataset on Jun 7, a total of 1034 confirmed and 72 suspected cases have been reported in 39 countries or regions [7] (Additional file 1: Table S1). No related deaths have been reported so far [6]. Importantly, the World Health Organization (WHO) emphasizes that there is a widespread human-to-human transmission among the population, and the virus may have been circulating insidiously for a period of time [6].

Unlike the previous sporadic cases, the current outbreak has occurred in human populations with no travel links to an endemic area of Africa. It is puzzling which factors are driving and helping the unprecedented spread of MPXV. Some possible reasons listed as follows may explain the unusual outbreak. (1) The declining population immunity against smallpox plays a substantial role in the reemergence of monkeypox. Currently, a tremendous human population has no immunity against smallpox, monkeypox and other orthopoxvirus infections after the 30-year cessation of the smallpox vaccination. During this outbreak, high prevalence was observed among adults aged 21–40 years without the smallpox vaccination. This situation may allow the continuous circulation of MPXV in the human populations and consequently alter the susceptibility of the virus to humans. (2) The emergence of new transmission patterns may facilitate the spread of the MPXV. In this outbreak, the MPXV has been transmitted in the population of men who have sex with men (MSM) through sexual activity and is spreading like a sexually transmitted disease. Close contact during sexual activity or intimate activities, including prolonged skin-to-skin contact, may be essential in the human-to-human transmission. However, further research is required to clarify whether monkeypox can be defined as a sexually transmitted disease. Another explanation for the unexpected men-to-men transmission is that the MPXV was introduced into the MSM cluster coincidentally and continued spreading among them [8]. (3) Mutations in the MPXV may enhance its transmissibility. Although the MPXV is a DNA virus with a lower mutation rate, the possibility should be included that under certain selective pressure, adaptive mutations of the MPXV are likely to accumulate. The latest analysis indicated that the MPXVs in this outbreak show single nucleotide mutations and frameshift mutations compared with previous ones [9, 10]. Whether there is a genetic basis for the unprecedented viral spread outside of Africa needs to be characterized precisely to explain this possibility.

This outbreak may reinforce the monkeypox as a novel and urgent threat after COVID-19. There have been no extensive studies on the disease since it was reported. Therefore, clinicians and scientists should pay attention to the potential biosafety issues involved in this disease. Our understanding of MPXV epidemiology, biology, ecology, and pathophysiology has demonstrated some important gaps. While rodents are regarded as the natural reservoir of the MPXV, the natural history of the virus is not entirely known, which prevents the identification of potential sources of zoonotic transmission. Further epidemiological studies and whole-genome sequencing of biological samples may help identify the origin responsible for this outbreak. In addition, it is necessary to clarify viral, immunological, and pathological mechanisms underlying MPXV infection. Furthermore, with the increasing number of cases occurring globally, vaccines and drugs specific to monkeypox need to be developed. Rapid diagnosis in potential patients is necessary for better disease management. As recently recommended by the WHO, monkeypox should be actively monitored and extensively studied worldwide. Therefore, it is also necessary to investigate and assess the risk of the disease pandemic. Clinicians must be alert to any rash with a presentation similar to monkeypox and carefully identify monkeypox from vesicular herpes for differential diagnosis. Professional training and awareness among clinicians and the development of disease management guidelines are crucial to improving the diagnosis and treatment of monkeypox. Moreover, recommendations and advice are needed for people about the measures they can take to prevent infection, such as careful hand-washing and mask-wearing. Furthermore, a supply of reagents for diagnosis, prevention and treatment against the virus should be maintained as a precaution.

In summary, the increasing prevalence of monkeypox is likely to be a new potential threat globally in the future. Recently, more intensive surveillance and investigation on the MPXV are urgently needed to contain the spread of the virus. Finally, the epidemic may be brought under control with significant public health and medical efforts.

## Supplementary Information

**Additional file 1**: **Fig. S1** Timeline of major events in this monkeypox outbreak from May 7th to May 30th, 2022. **Table S1** Distribution of reported monkeypox cases worldwide until May 30th, 2022.

## Data Availability

Not applicable.

## Abbreviations

COVID-19: Coronavirus disease 2019
MPXV: Monkeypox virus
MSM: Men who have sex with men
UK: United Kingdom
UKHSA: UK Health Security Agency
WHO: World Health Organization
