# Supplementary material for: Monkeypox outbreak: A novel threat after COVID-19?
Source: Mil Med Res. 2022 Jun 13;9:29. doi: 10.1186/s40779-022-00395-y (PMC9190192; doi:10.1186/s40779-022-00395-y)
Supplement: Supplementary file 1 — Additional file 1: Fig. S1 Timeline of major events in this monkeypox outbreak from May 7th to May 30th, 2022. Table S1 Distribution of reported monkeypox cases worldwide until May 30th, 2022. [file 40779_2022_395_MOESM1_ESM.pdf]

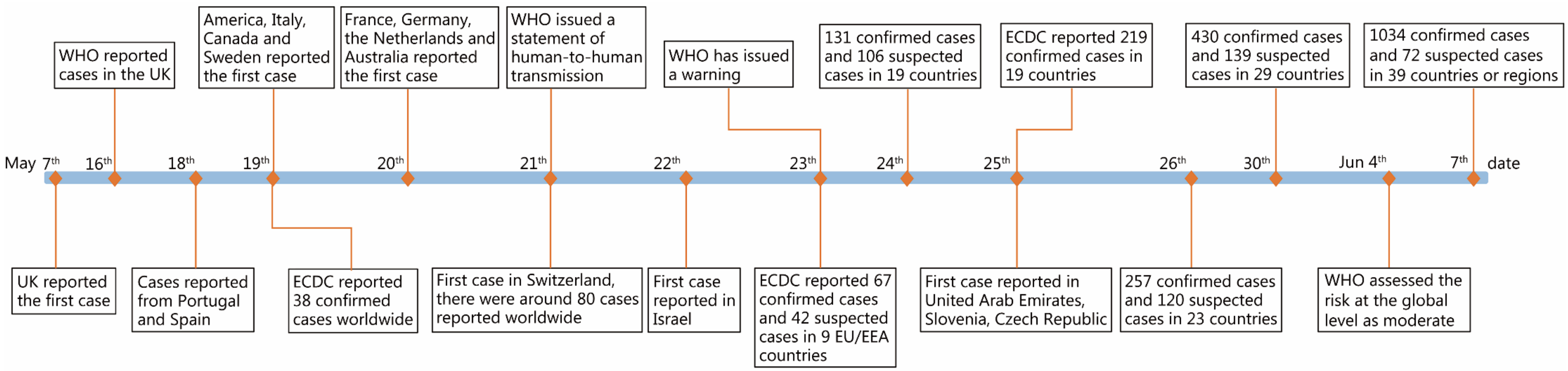

Fig. S1 Timeline of major events in this monkeypox outbreak from May 7th to Jun 7th, 2022. ECDC European Centre for Disease Prevention and Control, UK United Kingdom, WHO World Health Organization

Table S1 Distribution of reported monkeypox cases worldwide until Jun 7th, 2022

	Country/Region	Confirmed case number	Suspected case number
Europe	United Kingdom	303	0
	Spain	198	32
	Portugal	153	0
	Germany	80	0
	France	52	0
	Netherlands	40	0
	Italy	25	2
	Belgium	17	0
	Czech Republic	6	0
	Switzerland	8	0
	Sweden	5	0
	Denmark	2	0
	Ireland	6	0
	Austria	1	0
	Finland	2	0
	Slovenia	3	0
	Norway	2	0
	Hungary	1	0
	Latvia	1	0
	Malta	1	0
	Kosovo	0	1
North America	Canada	81	14
	United States	27	2
	Mexico	1	0
	Haiti	0	1
	Cayman Islands	0	1
South America	Argentina	2	0
	Bolivia	0	3
	French Guiana	0	2
	Brazil	0	6
	Paraguay	0	1
	Uruguay	0	1
Asia	United Arab Emirates	8	0
	Israel	2	0
	Iran	0	3
	Pakistan	0	1
Africa	Morocco	1	0
	Sudan	0	1
Oceania	Australia	6	1
Total		1034	72
